# Five common tumor biomarkers and CEA for diagnosing early gastric cancer: A protocol for a network meta-analysis of diagnostic test accuracy

**DOI:** 10.1097/MD.0000000000010577

**Published:** 2018-05-11

**Authors:** Minghui Shen, Hui Wang, Kongyuan Wei, Jianling Zhang, Chongge You

**Affiliations:** a Department of Clinical Laboratory, Second Hospital of Lanzhou University; b Department of General Surgery, First Affiliated Hospital of Lanzhou University, Lanzhou, China.

**Keywords:** CEA, gastric cancer, network meta-analysis, sensitivity and specificity, tumor biomarkers

## Abstract

**Background:**

Although surgical resection is the recommended treatment for the patients with gastric cancer, lots of patients show advanced or metastatic gastric cancer at the time of diagnosis. Detection of gastric cancer at early stages is a huge challenge because of lack of appropriate detection tests. Unfortunately, existing clinical guidelines focusing on early diagnosis of gastric cancer do not provide consistent and prudent evidence. Serum carcinoembryonic antigen was considered as a complementary test, although it is not good enough to diagnose early gastric cancer. There are no other tumor markers recommended for diagnosing early gastric cancer. This study aims to evaluate and compare the diagnostic accuracy of 5 common tumor biomarkers (CA19–9, CA125, PG, IncRNA, and DNA methylation) and CEA and their combinations for diagnosing gastric cancer through network meta-analysis method, and to rank these tests using a superiority index.

**Methods:**

PubMed, EMBASE.com, and the Cochrane Central Register of Controlled Trials (CENTRAL) will be searched from their inception to March 2018. We will include diagnostic tests which assessed the accuracy of the above-mentioned tumor biomarkers and CEA for diagnosing gastric cancer. The risk of bias for each study will be independently assessed as low, moderate, or high using criteria adapted from Quality Assessment of Diagnostic Accuracy Studies 2 (QUADAS-2). Network meta-analysis will be performed using STATA 12.0 and R 3.4.1 software. The competing diagnostic tests will be ranked by a superiority index.

**Results:**

This study is ongoing and will be submitted to a peer-reviewed journal for publication.

**Conclusion:**

This study will provide systematically suggestions to select different tumor biomarkers for detecting the early gastric cancer.

## 1 Introduction

Gastric cancer is the most common tumor in the gastrointestinal carcinomas.^[1]^ It is the fifth leading cause of cancer death in the world.^[2]^ Although with mounts of effort in the management of gastric cancer, the number of patients with gastric cancer is increasing and gastric cancer is now influencing the healthy conditions of people more and more broadly.^[3]^

Despite surgical resection is a recommended treatment, chemotherapy has been proved to be safe and efficient to prolong survival in the patients with gastric cancer.^[4]^ However, there are still lots of patients with advanced or metastatic gastric cancer when they got diagnosis.^[5]^ So, it is still a huge challenge to detect gastric cancer at early stages because of vacancy of specific detection tests.^[6]^

There have been lots of investigations to find the accurate serum and tumor biomarkers to detect gastric cancer. Now, some tumor biomarkers (such as LncRNA, CA125, CA19–9, etc) have been applied for gastric cancer detection, while the clinical applicability of the tests is not very clear.^[7]^ Unfortunately, existing clinical guidelines focusing on early diagnosis of gastric cancer do not provide consistent and prudent evidence. Serum carcinoembryonic antigen was considered as a complementary test, although it is not good enough to diagnose early gastric cancer.^[8]^ Besides, there are not recommended tumor biomarkers to diagnose early gastric cancer.^[9]^

Gastric cancer screening in the future might be relied on tumor biomarkers. A combination of serum CEA and CA19–9 has been indicated to obtain higher specificity than serum CEA alone^[10]^; the combination of CEA, CA125, and CA19–9 has been reported to attain higher sensitivity than CEA alone.^[11]^ What is more, the combination CEA and LncRNA was more accurate ^[12]^ and the combination of CEA and PG was proved to improve sensitivity.^[13]^ Recent studies ^[14–16]^ showed that DNA methylation was a novel biomarker for diagnosis of early gastric cancer and the combination of CEA and DNA methylation might be more efficient. However, according to the recent studies, it does not remain clear which individual test or combined test is beneficial to detect gastric cancer at early stages.

Network meta-analysis has been considered to extend conventional meta-analyses on multiple treatments (i.e., 3 or more) for a given condition.^[17]^ Hence, there may be multiple candidate tests for diagnosing a particular disease outcome in a diagnostic test accuracy study.^[18]^ In order to present an overall picture, network meta-analysis (mainly refers to indirect comparison) has been proposed by some researchers to simultaneously compare the accuracy of multiple tests within and between studies and rank the diagnostic tests using diagnostic odds ratio (DOR) and a superiority index.^[18–23]^

This study aims to evaluate and compare the accuracy of 5 common tumor biomarkers (CA19–9, CA125, PG, IncRNA, and DNA methylation) and CEA and their combinations for diagnosing gastric cancer through network meta-analysis method and to rank these tests using superiority index.

## 2 Methods

### 2.1 Design and registration

A network meta-analysis of diagnostic test accuracy will be conducted. We have registered the protocol on the international prospective register of systematic review (PROSPERO).^[24]^ We will follow the Preferred Reporting Items for Systematic Reviews and Meta-Analyses (PRISMA) ^[25]^ statements for reporting our systematic review.

### 2.2 Information sources

We will search PubMed, EMBASE, and the Cochrane Central Register of Controlled Trials (CENTRAL) until March 2018. The search strategies will be conducted by SMH and WH who are experienced information specialists. We will search the references of relevant systematic reviews/meta-analyses to identify additional potential studies.

### 2.3 Search strategy

The search terms will include gastric neoplasm, stomach neoplasms, stomach neoplasm, stomach cancers, stomach cancer, gastric cancer, gastric cancers, CEA, carcinoembryonic antigen, sensitivity, and specificity.

Search strategy of PubMed was as follows:

#1 ((((((“gastric cancer”[MeSH Terms]) OR “gastric cancers”[Title/Abstract]) OR “Gastric cancer”[Title/Abstract])OR “Gastric cancers”[Title/Abstract]) OR “Stomach cancer”[Title/Abstract]) OR “stomach cancers”[Title/Abstract])

#2 ((CEA [MeSH Terms]) OR carcinoembryonic antigen [Title/Abstract])

#3 ((((“CA199” [MeSH Terms]) OR “CA 199” [Title/Abstract]) OR “CA-199” [Title/Abstract]) OR “carbohydrate antigen 199” [Title/Abstract])

#4 ((((“CA125” [MeSH Terms]) OR “CA 125” [Title/Abstract]) OR “CA-125” [Title/Abstract]) OR “carbohydrate antigen 125” [Title/Abstract])

#5 ((“PG” [MeSH Terms]) OR “pepsinogen” [Title/Abstract])

#6 ((“IncRNA” [MeSH Terms]) OR “Inc RNA” [Title/Abstract])

#7 ((“DNA methylation” [MeSH Terms]) OR “deoxyribonucleic acid methylation ” [Title/Abstract])

#8 #2 AND #3

#9 #2 AND #4

#10 #2 AND #5

#11 #2 AND #6

#12 #2 AND #7

#13 #8 OR #9 OR #10 OR #11OR #12

#14 #1 AND #13

### 2.4 Eligibility criteria

#### 2.4.1 Type of study

Eligibility criteria are as follows: ① index tests include either CEA, CA125, CA19–9, PG, LncRNA, and DNA methylation, or combinations thereof; ② at least 2 index tests per study, one of them being CEA; ③ report or provide sufficient information to allow us to calculate the true positive (TP), false-positive (FP), true negative (TN), and false-negative (FN) values; ④ case–control, cross-sectional, or cohort designs; ⑤ there will be no limitations on language of publication, year of publication, publication status, or stage of gastric cancer.

#### 2.4.2 Patients

We will include studies that contain patients performed on CEA or CA199 or CA125 or PG or LncRNA or DNA methylation to predict malignant potential of gastric cancer. We will exclude studies that provide no sufficient data of diagnostic accuracy. We will put no limitations in age, gender, and nations.

#### 2.4.3 Index tests

We will consider CEA, CA199, CA125, PG, LncRNA, and DNA methylation as index tests because these tests are usually used to predict malignant potential of gastric cancer.

#### 2.4.4 Reference standards

Definitive histopathology following surgery will be considered as primary reference standard and the clinical follow-up after treatment will be the complementary reference standard.

#### 2.4.5 Study selection and data extraction

Primary search records will be imported into ENDNOTE X6 literature management software, according to eligibility criteria; we will screen the titles and abstracts of records to identify potential trials. We will obtain and review full-text versions of all potentially relevant trials to ensure eligibility.

We will use Microsoft Excel 2013 (Microsoft Corp, Redmond, WA, www.microsoft.com) to collect data, which include eligible studies characteristics (e.g., name of first author, year of publication, country in which the study was conducted, gold standard, index tests), patients characteristics (male, mean age, sample, method, cutoff level, risk factors of pancreatic cancer), and outcomes (sensitivity (SEN), specificity (SPE), TP, FP, FN, TN).

Study selection and data extraction will be conducted by 1 reviewer (SMH), and will be checked by other reviewers (WH, WKY). Any conflicts will be resolved by having a discussion.

#### 2.4.6 Quality evaluation

Two reviewers (SMH, WH) will independently evaluate the risk of bias for each study as low, moderate, or high using criteria adapted from Quality Assessment of Diagnostic Accuracy Studies 2 (QUADAS-2),^[26]^ and conflicts will be resolved by having a discussion.

#### 2.4.7 Geometry of the network

Network plots will be performed using R software version 3.4.1. In network plots, the size of the nodes is proportional to the number of studies evaluating a test, and thickness of the lines between the nodes is proportional to the number of direct comparisons between tests. The network is connected because there is at least 1 study evaluating a given test together with at least 1 of the other remaining tests.^[18]^ A loop connecting 3 tests indicates that there is at least 1 study comparing the 3 targeted tests simultaneously.^[27]^

#### 2.4.8 Network meta-analysis

##### 2.4.8.1 Pairwise meta-analyses

Pairwise meta-analyses will be performed for pooled SEN, SPE, positive likelihood ratio (PLR), negative likelihood ratio (NLR), DOR, and area under the summary receiver operating characteristic curve (AUSROC) using bivariate mixed-effects regression modeling with STATA version 12.0 (Stata, College Station, TX). The between-study variance will be calculated using var logitSEN and logitSPE.^[28,29]^ The proportion of heterogeneity according to the threshold effect among the included studies will be calculated by the squared correlation coefficient estimated from the between-study covariance variable in the bivariate model.^[30]^ The heterogeneity between each study will be estimated using the Q value and the inconsistency index (*I*^2^) test, and the values of 25%, 50%, and 75% for the *I*^2^ will be indicative of low, moderate, and high statistical heterogeneity, respectively.^[31]^

We will plan subgroup analyses for each biomarker on the basis of the country in which the study was conducted, stage of gastric cancer, cutoff level, risk factors of gastric cancer, and risk of bias.

We will perform the Deek funnel plot to evaluate the potential publication bias when there are more than 10 studies available for an index test.^[32]^

##### 2.4.8.2 Indirect comparisons between competing diagnostic tests

CEA is considered as common reference test and we will calculate relative diagnostic outcomes between index tests by analysis of variance (ANOVA) model in R software version 3.4.1,^[18]^ including relative sensitivity (RSEN), relative specificity (RSPE), and relative diagnostic odds ratio (RDOR).

##### 2.4.8.3 Ranking of competing diagnostic tests

As an attractive feature of network meta-analysis, ranking of interventions is more and more popular, but it is still a challenge to rank competing diagnostic tests. Some researchers regard DOR as an indicator of ranking of competing diagnostic tests, ^[22]^ while the measure might not distinguish between tests with high sensitivity but low specificity or vice versa. Besides, the superiority index introduced by Deutsch et al ^[23]^ provides more weight to tests performing relatively well on both diagnostic accuracy measures and less weight on tests performing poorly on both diagnostic measures or tests performing better on one measure but poorly on the other.^[18]^ The superiority index ranges from 0 to ∞, and tends toward ∞ and 0, as the number of tests to which the target test is superior and inferior increases, respectively, and the superiority index tending to 1 indicates that the tests are equal.^[18]^

## 3 Discussion

To the best of our knowledge, this will be the first diagnostic network meta-analysis comprehensively comparing different tumor biomarkers combined with or without CEA for gastric cancer. We hope that the results of the study will help clinicians and patients to select appropriate diagnostic test for pancreatic cancer.

## Author contributions

Planned and designed the research: SMH, WH, and WKY.

Tested the feasibility of the study: SMH, WH, and ZJL.

Provided methodological advice, polished and revised the manuscript: SMH, WH, and YCG.

Wrote the manuscript: SMH and YCG.

All authors approved the final version of the manuscript.

**Data curation:** Hui Wang, Kongyuan Wei.

**Methodology:** Hui Wang, Jianling Zhang.

**Software:** Kongyuan Wei, Jianling Zhang.

**Validation:** Jianling Zhang.

**Writing – original draft:** Minghui Shen, Chongge You.

**Writing – review & editing:** Minghui Shen, Chongge You.
